# callsync: An R package for alignment and analysis of multi‐microphone animal recordings

**DOI:** 10.1002/ece3.11384

**Published:** 2024-05-23

**Authors:** Simeon Q. Smeele, Stephen A. Tyndel, Barbara C. Klump, Gustavo Alarcón‐Nieto, Lucy M. Aplin

**Affiliations:** ^1^ Cognitive & Cultural Ecology Research Group Max Planck Institute of Animal Behavior Radolfzell Germany; ^2^ Department of Human Behavior, Ecology and Culture Max Planck Institute for Evolutionary Anthropology Leipzig Germany; ^3^ Department of Biology University of Konstanz Constance Germany; ^4^ Department of Ecoscience Aarhus University Aarhus Denmark; ^5^ Department of Behavioral and Cognitive Biology University of Vienna Vienna Austria; ^6^ Department of Migration Max Planck Institute of Animal Behavior Radolfzell Germany; ^7^ Department of Evolutionary Biology and Environmental Studies University of Zurich Zurich Switzerland; ^8^ Division of Ecology and Evolution, Research School of Biology The Australian National University Canberra Australian Capital Territory Australia

**Keywords:** bio acoustics, communication networks, microphone alignment, recording processing

## Abstract

To better understand how vocalisations are used during interactions of multiple individuals, studies are increasingly deploying on‐board devices with a microphone on each animal. The resulting recordings are extremely challenging to analyse, since microphone clocks drift non‐linearly and record the vocalisations of non‐focal individuals as well as noise. Here we address this issue with callsync, an R package designed to align recordings, detect and assign vocalisations to the caller, trace the fundamental frequency, filter out noise and perform basic analysis on the resulting clips. We present a case study where the pipeline is used on a dataset of six captive cockatiels (*Nymphicus hollandicus*) wearing backpack microphones. Recordings initially had a drift of ~2 min, but were aligned to within ~2 s with our package. Using callsync, we detected and assigned 2101 calls across three multi‐hour recording sessions. Two had loud beep markers in the background designed to help the manual alignment process. One contained no obvious markers, in order to demonstrate that markers were not necessary to obtain optimal alignment. We then used a function that traces the fundamental frequency and applied spectrographic cross correlation to show a possible analytical pipeline where vocal similarity is visually assessed. The callsync package can be used to go from raw recordings to a clean dataset of features. The package is designed to be modular and allows users to replace functions as they wish. We also discuss the challenges that might be faced in each step and how the available literature can provide alternatives for each step.

## Graphical abstract

To better understand how vocalisations are used during interactions of multiple individuals, studies are increasingly deploying on‐board devices with a microphone on each animal. To handle the long un‐aligned recordings, we present callsync, an R package designed to align recordings, detect and assign vocalisations to the caller, trace the fundamental frequency, filter out noise and perform basic analysis on the resulting clips.

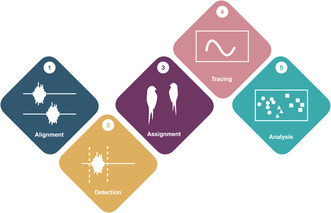

## 1 INTRODUCTION

The study of vocal signals in animals is a critical tool for understanding the evolution of vocal communication (Endler, 1993). Vocalisations commonly occur in group contexts, where they may function to signal movement or identity, and mediate interactions. However, the problem of assigning identity has led most previous work to focus on long‐range calls or on vocalisations made when alone, for example, territorial calls and song, and to focus on recording one individual at a time. Yet only by studying the ways that animals communicate in ‘real time’ can allow us to untangle the complicated dynamics of how group members signal one another (Gill et al., 2015). For example, the context of how individuals address members of their social network (Cheney & Seyfarth, 2018), and the ensuing call and response‐dynamics (Araya‐Salas et al., 2020), can only be studied by recording multiple individuals simultaneously. These communication networks can help us understand how animals coordinate call‐response with movement (Demartsev et al., 2023) as well as the function of vocal imitation (Dahlin et al., 2014; Knörnschild et al., 2012; Nousek et al., 2006).

Recent innovations in recording technologies have allowed for a dramatic increase of fine‐scale on‐animal bioacoustic data collection (Gill et al., 2016; Sanchez et al., 2021; Wild et al., 2022), allowing multiple individuals to be recorded simultaneously. However, as the capability of placing small recording devices on animals increases, so does the need for tools to process the resulting data streams. Several publicly available R packages exist that measure acoustic parameters from single audio tracks (*seewave*: Sueur et al., 2008; *tuneR*: Ligges et al., 2022; *WarbleR*: Araya‐Salas & Smith‐Vidaurre, 2017), but to our knowledge, none addresses the critical issue of microphone clock drift and the ability to align and process multiple recordings. This poses a serious issue for those studying communication networks of multiple tagged individuals. In this paper, we apply a new R package, callsync, that aligns multiple misaligned audio files, detects vocalisations, assigns these to the vocalising individual and provides an analytical pipeline for the resulting synchronised data.

**FIGURE 1:**
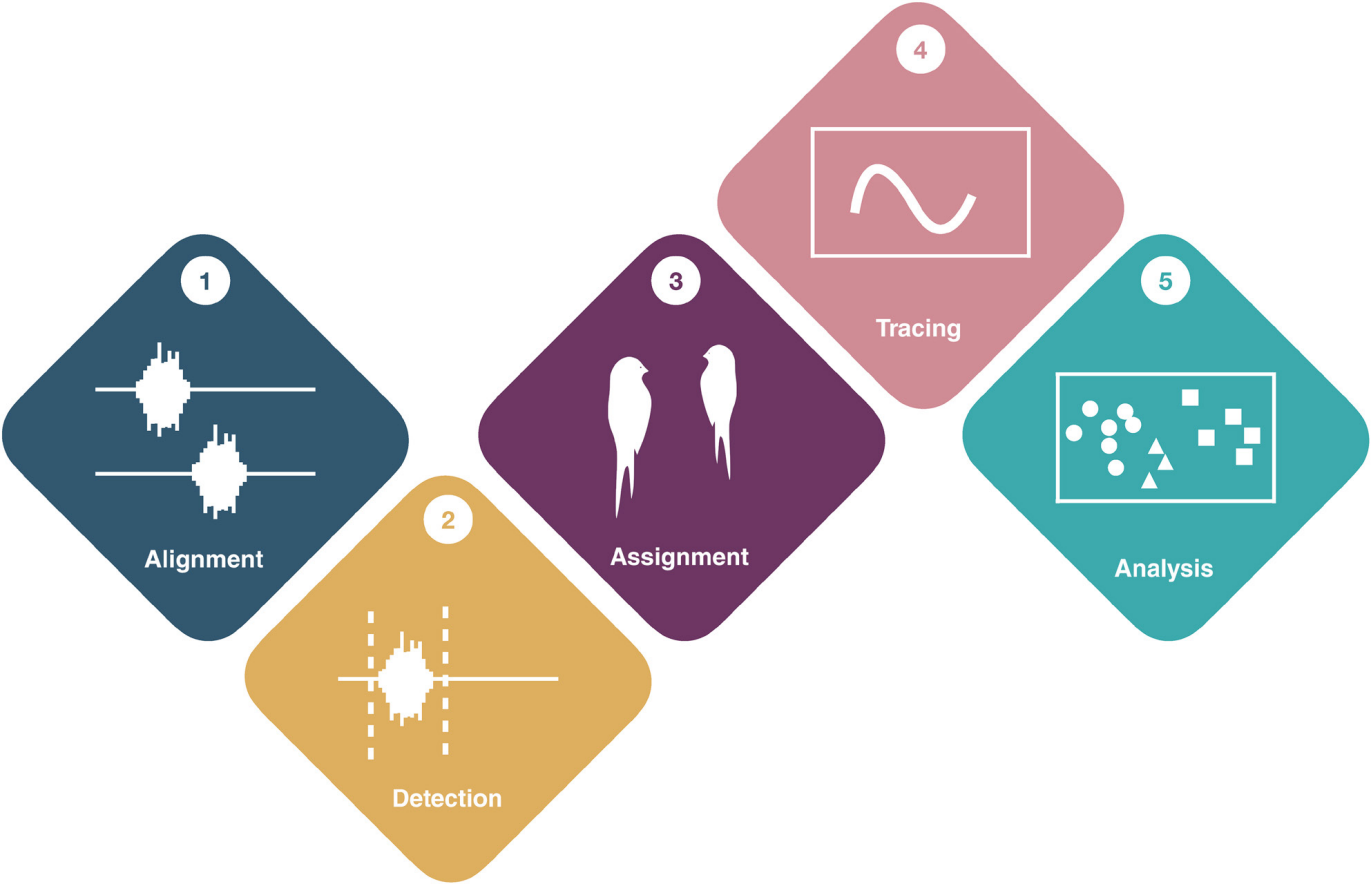
Flowchart from the callsync package. (1) The *alignment* module can be used to align multiple microphones that have non‐linear temporal drift. (2) The *detection* module can be used to detect vocalisations in each recording. Filters can be applied to remove false positives in the detection module. (3) The *assignment* module can be used to assign a vocalisation to the focal individual, making sure that vocalisations from conspecifics are excluded from the focal recording. (4) The *tracing* module can be used to trace and analyse the fundamental frequency for each vocalisation. (5) The final *analysis* module can be used to run spectrographic cross‐correlation and create a feature vector to compare across recordings.

The primary target for use of this package are researchers that study animal communication systems within groups. As researchers deploy multiple microphones that record simultaneously, the resulting clock drift can prove to be a barrier in further data processing steps (Schmid et al., 2010). To make matters worse this drift is often non‐linear (Anisimov et al., 2014). Thus, if several microphone recorders are placed on animals (whales: Hayes et al., 2000; Miller & Dawson, 2009; bats: Stidsholt et al., 2019), it is critical for researchers to be able to line up all tracks so that calls can be assigned correctly to the vocalising individual (loudest track). The main functionality of callsync is to align audio tracks, detect calls from each track, determine which individual (even ones in relatively close proximity to one another) is vocalising and segment them (detect start and end of the signal as in the detection module), as well as take measurements of the given calls (see Figure 1). As callsync takes a modular approach to aligning, detecting and analysing audio tracks, researchers can choose to use only the components of the package that suit their needs.

Current research packages that implement call alignment strategies are either used in Matlab (Anisimov et al., 2014; Malinka et al., 2020) or C++ (Gill et al., 2015). However, these tools have not, up to now, been adapted for the R environment, a popular programming language among many animal behaviour and bioacoustic researchers. Many of these tools are not documented publicly nor open source, and can require high licencing fees (i.e., Matlab). While the design of our package is best suited to contexts where all microphones exist in the same spatial area, it is the goal that it can be adapted to other contexts. callsync is publicly available on CRAN and GitHub, is beginner friendly with strong documentation and does not require extensive programming background. This open‐source tool will allow researchers to expand the study of bioacoustics and solve an issue that impedes detailed analysis of group‐level calls.

## 2 GENERATE EXAMPLE: SPRING PEEPER

We downloaded two Spring Peeper (Pseudacris crucifer) recordings from the Macaulay Library at the Cornell Lab of Ornithology (ML397067 and ML273028). Using these two recordings, we generated two artificial audio tracks, one drifted approximately 18 s from the other. Drift was simulated by inserting 0.03 s of background noise every second in one of the tracks. Background noise for each track was the same, and constructed by concatenating random snippets of noise from each of the Macaulay Library recordings. The background noise was then amplified by 4 dB.

Both artificial tracks incorporated 10 calls from each Macaulay recording. The calls were arranged to simulate alternating calling behaviour between the two frogs. Using the *tuneR* package (Ligges et al., 2022), we normalised the calls to simulate the presence of a focal individual, setting the focal calls to 85% and background calls to 55% of their maximum amplitude.

We then used callsync to align the tracks using 2 min chunks, and showed that all detected calls were assigned to the correct individual. We also showed that the fundamental frequency could be correctly traced. For details see the vignette in the CRAN version of callsync.

## 3 CASE STUDY: COCKATIELS

We present a case study to show how callsync functions can be included in a workflow (see Figure 1). We used a dataset of domestic cockatiels (*Nymphicus hollandicus*). These birds are part of an ongoing study at the Max Planck Institute of Animal Behaviour in Radolfzell, Germany. Thirty birds were housed in five groups of six individuals, with each group of six housed separately in a 4 × 3 × 2.7 m aviary facility. Each bird was fitted with a TS‐systems EDIC‐Mini E77 tag inside a sewn nylon backpack fitted via Teflon harness around the wings, with the total weight of all components under 7% of body weight (weight range of birds 85–120 g). Audio recordings were scheduled to record for 4 h per day. Each microphone was automatically programmed to turn on and off daily at the same time. For the purposes of demonstration, three full recording sessions (ca. 4 h) were selected for processing where the microphones were scheduled to record starting at approximately sunrise (July 15–16, 2021, and December 8, 2022). Two of the recording sessions (2021) included manually played beeps in the background; one every hour at 10 kHz, one every 10 min at 0.4 kHz. This was done to ensure that the alignment would work both with and without external assistance, as some circumstances for research might allow such an inclusion, while others may prohibit it. Seven days after deployment, microphone recorders were removed and recordings were downloaded as .wav files directly onto a computer from the tag, according to the manufacturer protocols. Data from each microphone were placed into the appropriate folder (see workflow instructions) and processed in accordance with our package workflow.

### 3.1 Installation and set‐up

The package call be installed from CRAN running install.packages('callsync') or a developmental version can be installed from GitHub (https://github.com/simeonqs/callsync):
**install.packages**('devtools')
**library**(devtools)
devtools**::install_github**('simeonqs/callsync')



While all underlying code and functions from the CRAN repository can be found in the aforementioned GitHub repository, we have created a separate repository from which readers can follow the code from the case study: https://github.com/simeonqs/callsync_an_R_package_for_alignment_and_analysis_of_multi‐microphone_animal_recordings. All required packages are also automatically installed and loaded for the case study when running the 00_set_up.R script from this repository.

### 3.2 Alignment of raw recordings

The general goal of the alignment step is to shift unaligned recordings to a degree that will allow vocalisations to be processed further. While researchers' requirements on alignment precision will vary, the output of this function should allow researchers to be able to identify that vocalisations picked up across all microphones are identified as the same call on every microphone. It should be noted that recordings are still not perfectly aligned, since clock‐drift occurs within the chunks.

The raw recordings consisted of three wav files of ca. 4 h for six cockatiels (i.e., 18 files total). The backpack microphones have internal clocks that sync with the computer clock when connected via USB and automatically turn recordings on and off. However, these clocks drift in time both during the off period, creating start times that differ up to a few minutes, and also during the recording period, causing additional variable drift up to a minute in 4 h of recording for the microphone model we used. The function align can be used as a first step to align the audio recordings. To accurately align these tracks, the full audio files were automatically split into 15‐min chunks of recording to ensure that drift was reduced to mere seconds. This value can be adjusted depending on the amount of drift. The function selects one recording (focal recording) and aligns all the other recordings relative to the selected recording using cross correlation on the energy content (summed absolute amplitude) per time bin (option step_size), in our case 0.5 s was used. This value can also be adjusted, lower values allow for greater precision but higher computational load. The call detection and assignment step (see below) includes fine alignment and we therefore accepted a potential error of 0.5 s. There is also an option to store a log file that records the offset for each chunk.
**align**(chunk_size = 15, *# how long should the chunks be in minutes*
 step_size = 0.5, *# bin size for summing in seconds*
 path_recordings = 'ANALYSIS/DATA', *# where raw data is stored*
 path_chunks = 'ANALYSIS/RESULTS/chunks', *# where to store the chunks*
 keys_rec = **c**('_**\\**(', '**\\**)_'), *# how to recognise the recording in the path*
 keys_id = **c**('ASWMUX', '.wav'), *# how to recognise the individual/microphone in the path*
 blank = 15, *# how many minutes should be discarded before/after*
 *# detection*
 wing = 10, *# how much extra should be loaded for alignment in*
 *# minutes*
 save_pdf = TRUE, *# should a pdf be saved*
 save_log = TRUE) *# should a csv file with alignment times be saved*



**FIGURE 2:**
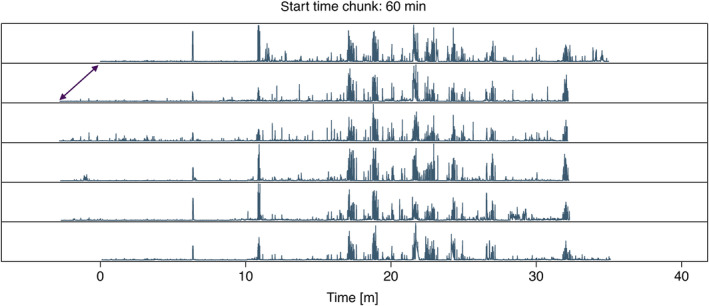
Example of the alignment output. Olive‐coloured lines represent the summed absolute amplitude per bin (= 0.5 s). Recordings are aligned relative to the first recording (which starts at 0). Note that recordings 2–5 initially started ~2 min earlier (purple arrow) but are now aligned. The title displays the start time of the chunk in the raw recording.

For cross correlation, the function align loads the chunks with additional minutes before and after (option wing) to ensure that overlap can be found. The cross correlation is performed using the function simple.cc, which takes two vectors (the binned absolute amplitude of the two recordings) and calculates the absolute difference while sliding the two vectors over each other. It returns the position of minimum summed difference, or in other words, the position of maximal overlap. This position is then used to align the recordings relative to the first recording and save chunks that are maximally aligned. Note that due to drift during the recording, the start and end times might still be seconds off; it is the overall alignment of the chunks that is optimised. The function also allows the user to create a pdf document with waveforms of each individual recording and a single page per chunk (Figure 2), to visually verify if alignment was successful. For our dataset all chunks but one aligned correctly without a filter. If this is not the case the option ffilter_from can be set to apply a high‐pass filter to improve alignment. Mis‐aligned chunks can also be rerun individually (option chunk_seq) to avoid re‐running the entire dataset. This was done for the case study as well (recording session from 16 July 2021, start time 15 min).

### 3.3 Call detection and assignment

The next step is to detect calls in each set of chunks and assign them to the correct individual. The detect.and.assign function wrapper loads the chunks using the function load.wave where it optionally applies a high‐pass filter to reduce the amount of low‐frequency noise. To detect calls, detect.and.assign first calls the function call.detect.multiple, which is used to detect multiple calls in an R wave object. It first applies the *env* function from the *seewave* (Sueur et al., 2008) package to create a smooth Hilbert amplitude envelope. It then detects all the points on the envelope which are above a certain threshold relative to the maximum of the envelope. After removing detections that are shorter than a set minimum duration (option min_dur) or longer than a set maximum (option max_dur) it returns all the start and end times as a data frame.

**FIGURE 3:**
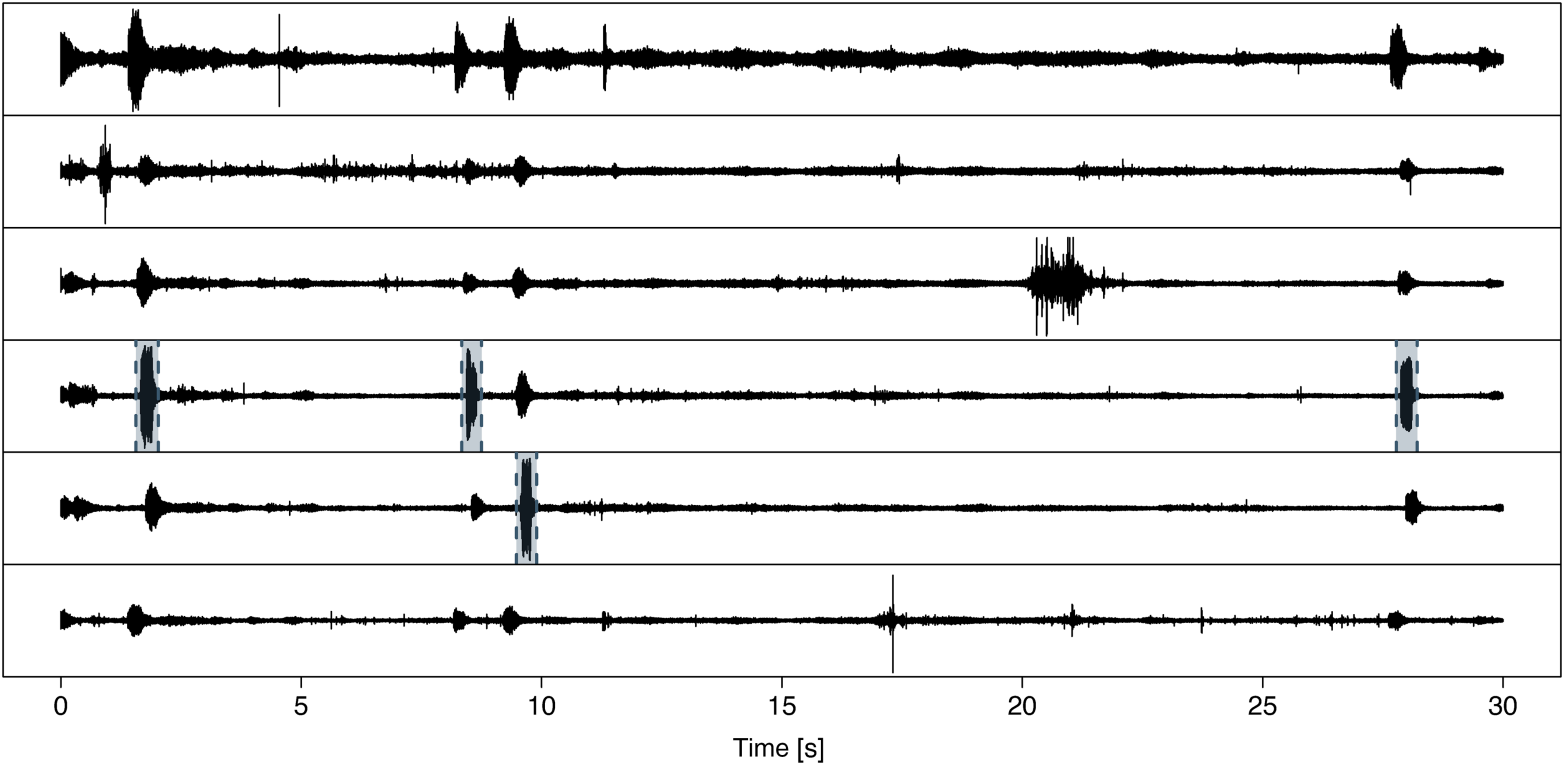
Example of the detection output. Black lines are the waveforms. Olive‐coloured dashed lines with shaded areas in between are the detected calls. Note that only the loudest version of each call is selected. The first call is also loud in the first channel (top‐row) but contains less energy throughout the whole call (channel four is loud for a longer duration). Animals with individual specific sounds (scratching, etc.) are seen clearly on only single microphones.

Because the microphones on focal individuals are very likely to record the calls of the non‐focal individuals as well, we implemented a step that assigns the detected calls to the individual emitting the sound, based on amplitude. For this, detect.and.assign subsequently calls the function call.assign, which runs through all the detections in a given chunk for a given individual (i.e., output of call.detect.multiple) and runs the call.detect function to more precisely determine the start and end time of the call. It then ensures that minor temporal drift after the align function is corrected by rerunning the simple.cc function pairwise between the focal recording and all others. After alignment it calculates the summed absolute energy content on all recordings for the time frame when the call was detected and compares the recording where the detection was made to all other recordings. If the loudest recording is louder by a set percentage (this value can be adjustable according to the researchers needs) than the second loudest recording, the detection is saved as a separate wav file. If not, this means it's not possible to determine the focal individual and the detection is discarded (there is an option to save all detections before the assignment step). The function also allows the user to create a pdf file with all the detections (see Figure 3 for a short example) to manually inspect the results.
**detect.and.assign**(path_chunks = 'ANALYSIS/RESULTS/chunks', *# where to read the chunks*
 path_calls = 'ANALYSIS/RESULTS/calls', *# where to store the calls*
 ffilter_from = 1100, *# from where to filter in Hz*
 threshold = 0.18, *# fraction of maximum of envelope for*
 *# detection*
 msmooth = **c**(1000, 95), *# smoothening argument for `env`*
 min_dur = 0.1, *# minimum duration in seconds for acceptance*
 max_dur = 0.3, *# maximum duration in seconds for acceptance*
 step_size = 1**/**50, *# bin size for summing in seconds*
 wing = 10, *# how many extra seconds to load for*
 *# alignment*
 save_files = TRUE, *# should the files be stored in path_calls*
 save_extra = 0.05) *# save 0.05 seconds before and after*
 *# detection*



For the 12‐h‐long cockatiel dataset, the function detected and assigned 4409 events, 2101 of which were retained as vocalisations (see next step). The removed events were primarily noise and noisy calls (e.g., overlapping calls and calls with echo). To estimate the accuracy of the function we manually detected and assigned IDs for 300 calls in three chunks (chosen due to high vocal activity), ran the same filtering step as callsync functions described earlier to get rid of 100 noisy examples, and compared the performance of the detect.and.assign function to manually labelled data. The ground truth was performed using the function calc.perf. This function simply determines whether the detected calls (and subsequent labelled IDs) of two datasets match. If sufficient temporal overlap exists between the two datasets, they are determined to match. The false positive rate was <4%, with noise (two detections) or noisy calls (four detections) being responsible; and the true positive rate was 81%. The remaining 19% (false negatives) were mostly calls that were too quiet to be picked up by our chosen amplitude threshold.

### 3.4 Tracing

**FIGURE 4:**
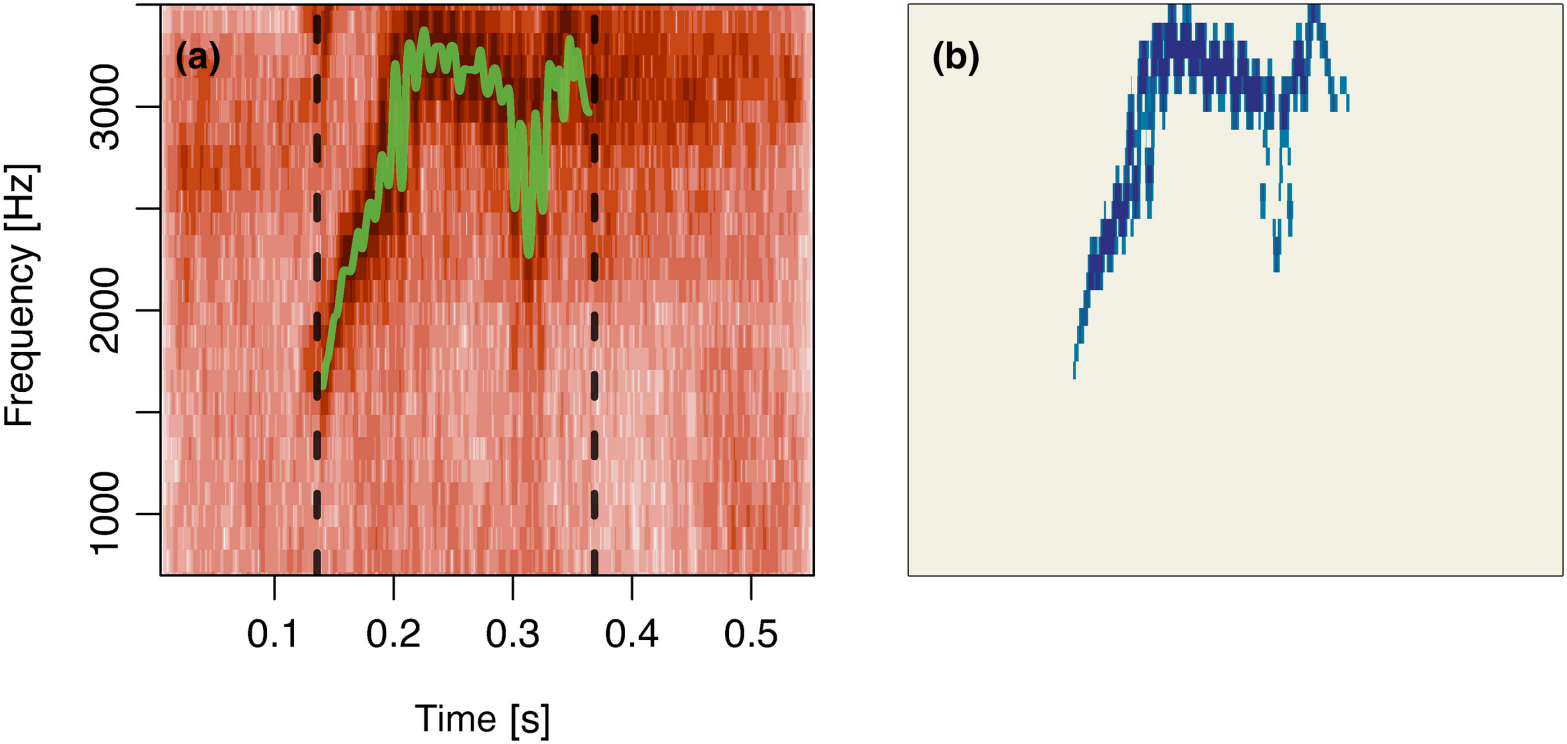
(a) Spectrogram of a cockatiel call with start and end (black dashed lines) and the fundamental frequency trace (green solid line). (b) Noise reduced spectrogram where darker colours indicate higher intensity.

To analyse the calls, the chunks were loaded and the function call.detect was run to determine the start and end times of the call. The wave objects were then resized to only include the call (new_wave). To trace the fundamental frequency we applied the trace.fund function to the resized wave objects. This function detects the fundamental frequency in a sliding window based on the spectrum and a relative threshold. We ran the latter step in parallel using the function *mclapply* from the base R package *parallel* (see Figure 4a for an example).
traces = **mclapply**(new_waves, **function**(new_wave) *# apply the function to each new_wave*
 **trace.fund**(wave = new_wave, *# use the new_wave*
 spar = 0.3, *# smoothing argument for the `smooth.spline` function*
 freq_lim = **c**(1.2, 3.5), *# only consider trace between 1.2 and 3.5 Hz *
 thr = 0.15, *# threshold for detection, fraction of max of spectrum*
 hop = 5, *# skip five samples per step*
 noise_factor = 1.5), *# only accept if trace is 1.5 times greater than noise*
 mc.cores = 4) *# run on four threads, has to be 1 on Windows*



Since the call detection step also picks up on a lot of noise (birds scratching, flying, walking around) as well as calls, we ran a final step to filter the measurements and traces before these were saved.
measurements = **measure.trace.multiple**(traces = traces, *# object containing traces*
 new_waves = new_waves, *# object containing adjusted waves*
 waves = waves, *# object containing waves*
 detections = detections, *# object containing detections*
 path_pdf = path_pdf_traces) *# where to store pdf*
keep = measurements**$**prop_missing_trace **<** 0.1 **&** *# max 10% missing points*
 measurements**$**signal_to_noise **>** 6 **&** *# signal to noise at least 6*
 measurements**$**band_hz **>** 400 **&** *# bandwidth at least 400 Hz*
measurements = measurements[keep,] *# keep only these measurements*
traces = traces[keep] *# and these traces*



### 3.5 Analysis

**FIGURE 5:**
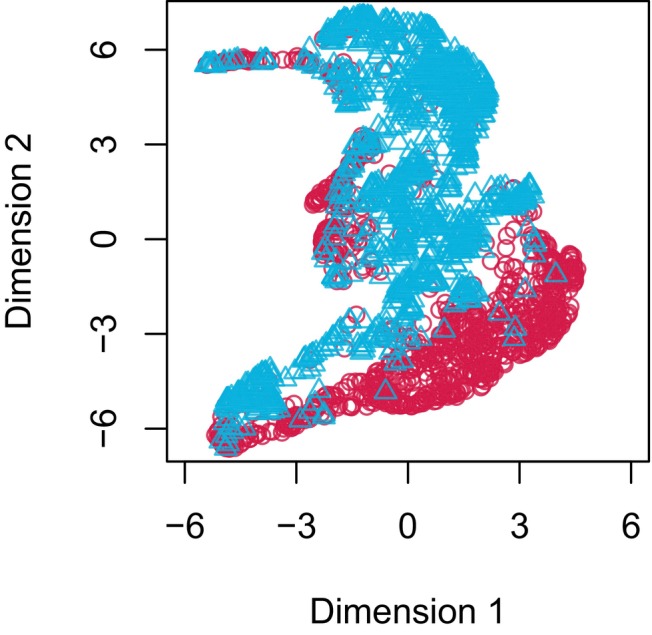
Call distribution in uniform manifold approximation and projection space. Dots represent calls and are coloured by year.

A frequently used method to compare calls is to measure their similarity using spectrographic cross correlation (SPCC) (Cortopassi & Bradbury, 2000), where two spectrograms are slid over each other and the pixelwise difference is computed for each step. At the point where the signals maximally overlap one will find the minimal difference. This score is then used as a measure of acoustic distance between two calls. The function run.spcc runs SPCC and includes several methods to reduce noise in the spectrogram before running cross‐correlation (for an example see Figure 4b). To visualise the resulting feature vector from running SPCC on the cockatiel calls we used uniform manifold approximation and projection (UMAP) (Konopka, 2022) which projects the results in two‐dimensional space. Calls cluster very strongly by the two separate recording groups (2021 and 2022), giving some evidence of different vocal signatures (see Figure 5). This is a very simple analysis, and we only included it to illustrate a potential use of the results from the previous steps.

## 4 DISCUSSION

Here we detail our R package callsync, designed to take raw microphone recordings collected simultaneously from multiple individuals and align, extract and analyse their calls. We present callsync performance on a computer‐generated dataset of 10 min of recording from two frogs and a case study of 12 h of natural recordings from two times six communally housed captive cockatiels. Each of the modular components (alignment, detection, assignment, tracing and analysis) successfully achieved the stated goals in both systems. In the computer generated dataset, no errors were made. In the case study, misaligned audio tracks were accurately aligned in a first step (see Figure 2), calls were correctly identified in the aligned recordings (see Figure 3), the individual making the call was selected (see Figure 3), and downstream data analysis was performed (Figures 4 and 5). callsync can perform alignment even on inter‐microphone drift that constitutes minutes as well as handle unpredictable and non‐linear drift patterns on different microphones.

With tracks aligned to a few seconds and only six false positives in the case study, we are confident that callsync is a robust and useful tool for bioacoustics research. Additionally, one false positive was actually a true positive; in this case because of a tiny difference in start and end time between the ground truth and automatic detection, which led the ground truth to be filtered out, but the automatic detection to stay. Overall, the true positive rate of our results was 81%, meaning that only 19% of the manually selected calls for ground truthing were not detected by the call.detect function. Where call rate across call types is important, researchers can set the threshold very low and manually remove false positives. Alternatively, a deep neural network can be used to sort signals from noise (Bergler et al., 2022).

A further possible challenge with the call.detect function could be that certain call types are never easily distinguishable from background noise. In these situations, call.detect is likely to pick up a significant amount of background noise in addition to calls. Function parameters can be adapted and should function on most call‐types, as can post‐processing thresholding. For example, machine learning approaches (Bergler et al., 2022; Cohen et al., 2022; Stowell et al., 2019) or image recognition tools (Smith‐Vidaurre et al., 2020; Valletta et al., 2017) can be later applied to separate additionally detected noise in particular circumstances where an amplitude based thresholding approach is insufficient. As well, in specific cases (e.g., low amplitude calls), once the align function is performed, the entire call.detect function can be swapped out for deep learning call detection algorithms such as ANIMAL‐SPOT (Bergler et al., 2022) or other signal processing approaches (e.g., *seewave* package Sueur et al. 2008).

The microphones used in this case study were implemented in a captive setting where all calls were within hearing range of each other and each microphone. Thus, all microphones contained a partially shared noisescape. Despite their proximity to one another, it should be noted that each microphone still contained unique vocal attributes, such as wing beats and scratching, and yet the major alignment step still aligned all chunks correctly. This was the case both in recordings with beep markers and without. It is possible that researchers find that the noise differences between microphones are too high for the first alignment step to perform adequately. This would be particularly salient in field settings where individuals within fission‐fusion groups find themselves in the proximity of other group members only some of the time (Balsby & Bradbury, 2009; Buhrman‐Deever et al., 2008; Furmankiewicz et al., 2011), or in situations where animals constantly move (i.e., flying) or do independent behaviours that other group members do not (e.g., preening) (Demartsev et al., 2023). Researchers will have to assess their own dataset and test this package to determine whether the first step will perform well on their dataset. If it does not, callsync is a modular package and other approaches, such as deep learning (O'shea & West, 2016) can be used instead of the first align function, while still using other components of the pipeline. Alternatively, beep markers audible on all microphones could be implemented to assist with alignment. Of course, the tones would need to be audible on all microphones.

Assigning a focal bird may be more difficult if individuals tend to be in very close proximity to other group members. If the group members spend time too close to one another (in our case less than 5–10 cm, the distance between the backpack and head of the vocalising individual) (Boughman & Wilkinson, 1998; Kloepper & Kinniry, 2018), it will become more difficult to distinguish the vocalising individual from other surrounding individuals. In the context of this case study, it was sufficient to only select a vocalising individual when the second loudest call was at least 5% quieter than the focal call. This threshold can be adapted if needed, or the assignment part can be removed entirely if so chosen. More generally, the issue of variation in spatial proximity could be solved by including other on‐board or group‐level tools, such as accelerometers (Anisimov et al., 2014; Gill et al., 2015), or proximity tags (Wild et al., 2022). This adds extra weight and cost to on‐board devices, and so if not possible, the other analytical tools could be considered to distinguish the focal calling birds. The specific tool will depend heavily on use‐cases, but include using discriminant analysis (McIlraith & Card, 1997) or cepstral coefficients (Lee et al., 2006) to predict the ID of the caller.

Lastly, both fundamental frequency tracing and SPCC work well in certain contexts. For example, automatic fundamental frequency traces work best for tonal calls while SPCC works best when the signal to noise ratio is sufficiently low (Cortopassi & Bradbury, 2000). However, if these criteria are not met, several other tools can be used to manually trace fundamental frequency instead, such as Luscinia (Lachlan et al., 2018) or manual tracing (Araya‐Salas & Smith‐Vidaurre, 2017). While it is important to consider all possible limitations of callsync it should also be noted that there are few tools that exist that perform this much‐needed task. Indeed, the fine‐scale alignment step of callsync allows for call and response dynamics to be measured regardless of how close the calls are to one another. While this paper has thus far only addressed on‐board microphones, other systems that implement passive acoustic monitoring systems (Thode et al., 2006) and microphone arrays (Blumstein et al., 2011) should also find benefit within this package depending on the set‐up and degree of drift. Possible future research opportunities include trying to incorporate machine learning and noise reduction techniques so that the major alignment can perform in all contexts.

## 5 CONCLUSION

This open‐source package is publicly available on GitHub and CRAN. We welcome all continued suggestions and believe that our package will result in an increase in the scope of bioacoustic research. Our package provides functions that allow alignment, detection, assignment, tracing and analysis of calls in a multi‐recorder setting where all microphones are within acoustic distance. The package can be used to generate a fully automated pipeline from raw recordings to the final feature vectors. We show that such a pipeline works well on a captive dataset with 4‐h‐long recordings from backpack microphones on six cockatiels which experience non‐linear time drift up to several minutes. Each module can also be replaced with alternatives and can be further developed. This package is, to our knowledge, the first R package that performs this task. We hope this package expands the amount of data researchers can process and contributes to understanding the dynamics of animal communication.

## AUTHOR CONTRIBUTIONS

**Simeon Q. Smeele:** Conceptualization (equal); data curation (equal); formal analysis (lead); investigation (equal); methodology (lead); project administration (lead); software (lead); validation (equal); visualization (lead); writing – original draft (supporting); writing – review and editing (equal). **Stephen A. Tyndel:** Conceptualization (equal); data curation (equal); formal analysis (supporting); investigation (equal); methodology (equal); project administration (supporting); software (supporting); validation (equal); visualization (supporting); writing – original draft (lead); writing – review and editing (equal). **Barbara C. Klump:** Conceptualization (supporting); supervision (equal); writing – review and editing (equal). **Gustavo Alarcón‐Nieto:** Investigation (equal); writing – review and editing (equal). **Lucy M. Aplin:** Conceptualization (supporting); investigation (supporting); resources (lead); supervision (equal); writing – review and editing (equal).

## CONFLICT OF INTEREST STATEMENT

The authors declare no conflicts of interest.

### OPEN RESEARCH BADGES

This article has earned Open Data and Open Materials badges. Data and materials are available at https://github.com/simeonqs/callsync_an_R_package_for_alignment_and_analysis_of_multi‐microphone_animal_recordings and (https://doi.org/10.17617/3.3XHTJA).

## Data Availability

All code associated with this paper is publicly available on GitHub (https://github.com/simeonqs/callsync_an_R_package_for_alignment_and_analysis_of_multi‐microphone_animal_recordings). All data and code are also publicly available on Edmond (https://doi.org/10.17617/3.3XHTJA). The callsync package can be installed from CRAN and a developmental version can be found on GitHub (https://github.com/simeonqs/callsync).
